# Antimicrobial Peptides Derived from Bacteria: Classification, Sources, and Mechanism of Action against Multidrug-Resistant Bacteria

**DOI:** 10.3390/ijms251910788

**Published:** 2024-10-08

**Authors:** Raynichka Mihaylova-Garnizova, Slavena Davidova, Yordan Hodzhev, Galina Satchanska

**Affiliations:** 1 Department of Natural Sciences, New Bulgarian University, Montevideo Blvd. 21, 1618 Sofia, Bulgaria; doctor.mihaylova@gmail.com (R.M.-G.); stdavidova@nbu.bg (S.D.); jordanqvo@gmail.com (Y.H.); 2 Department of Infectious Diseases, Military Academy, George Sofiiski Str. 3, 1606 Sofia, Bulgaria

**Keywords:** bacterial AMPs, bacteriocins, antibiotic resistance, modes of action, antibacterial mechanism

## Abstract

Antimicrobial peptides (AMPs) are short, usually cationic peptides with an amphiphilic structure, which allows them to easily bind and interact with the cellular membranes of viruses, bacteria, fungi, and other pathogens. Bacterial AMPs, or bacteriocins, can be produced from Gram-negative and Gram-positive bacteria via ribosomal synthesis to eliminate competing organisms. Bacterial AMPs are vital in addressing the increasing antibiotic resistance of various pathogens, potentially serving as an alternative to ineffective antibiotics. Bacteriocins have a narrow spectrum of action, making them highly specific antibacterial compounds that target particular bacterial pathogens. This review covers the two main groups of bacteriocins produced by Gram-negative and Gram-positive bacteria, their modes of action, classification, sources of positive effects they can play on the human body, and their limitations and future perspectives as an alternative to antibiotics.

## 1. Introduction

Certain bacteria produce bacterial antimicrobial peptides (AMPs) through the ribosomal pathway and are typically effective against closely related bacteria. In 1922, Alexander Fleming identified the first AMP and named it Lysozyme. After its discovery in 1928, Fleming also discovered the first antibiotic-penicillin. Florey, Chain, and Fleming brought the potential of penicillin in medical use into fruition and won the 1945 Nobel Prize in Medicine. In the 1940s and at the beginning of the golden age of antibiotics, the interest in using natural AMPs as therapeutics was lost. In the 1960s, interest in AMPs as host defense molecules was renewed due to the continuous rise of multidrug-resistant microbial pathogens. In 1981, α-helical AMPs named cecropins, isolated from *the Cecropia silk moth*, were discovered, followed by magainin from the African clawed frog *Xenopus laevis* in 1987. In the 1990s, the field of antimicrobial peptides expanded rapidly, reporting over 300 peptides. AMPs have been broadly identified and characterized in all living organisms [1].

Some bacteriocins exhibit a broad inhibitory range. The first bacteriocin, colicin, was identified in 1925 from an *Escherichia coli* strain. Many bacteriocins have been discovered since then, resulting in a diverse group of proteins in size, target, mode of action, delivery, and immunity mechanisms [2].

In 1939, René Dubos isolated gramicidin, a natural peptide from *Bacillus brevis* [3]. This peptide was effective against a broad spectrum of Gram-positive and Gram-negative bacteria and was the initial peptide-based topical antibiotic. Nevertheless, the enthusiasm for peptide drugs grew in the early 1980s following the isolation and characterization of cecropins and magainins [4]. 

**Figure 1:**
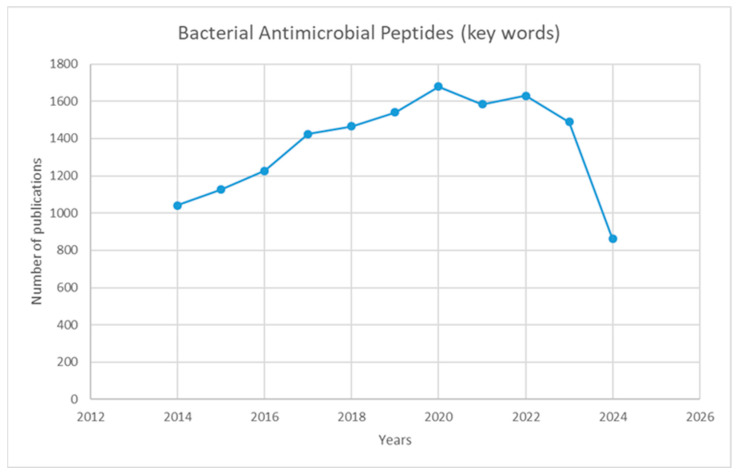
Graph showing the number of publications over ten years (2014–2024) using the keywords “bacterial antimicrobial peptides.” The search was done in July 2024, reaching 800 papers. They are expected to reach close to 1500 by the end of the year.

Antibiotic-resistant pathogens have become an urgent contemporary problem as many resistant strains continue to emerge [5]. Antimicrobial peptides can be considered alternative therapeutic agents and may be crucial for the fight against antibiotic resistance [6,7]. Figure 1 presents the dynamics of the total number of papers in the field for the last 10 years. The graphic shows the constant interest of researchers in AMPs, which has been accelerating since 2017.

**Figure 2:**
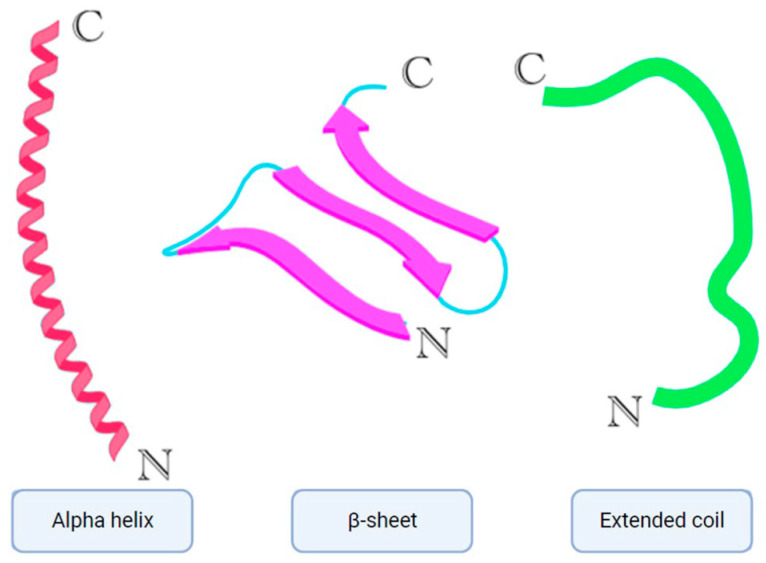
Simplified models of alpha helix, β-sheet, and extended coil structures [6].

Over 3000 AMPs have been contributed to the antimicrobial peptide database [8]. AMPs play a crucial role in the innate immune defense of organisms [9]. They are typically small molecules (10–100 amino acids) with an overall positive charge ranging from +2 to +9 and possess amphiphilic properties [10]. Depending on their target, AMPs are classified as antibacterial, antifungal, antiparasitic, and antiviral peptides [11]. The most prevalent AMPs are cationic peptides that exhibit a variety of secondary structures such as α-helices, β-sheets (Figure 2) with two or more disulfide bridges, loops with a single disulfide bond, and extended structures containing specific amino acids like proline, arginine, tryptophan, and glycine [12,13]. 

Bacteriocins have been identified and isolated from both Gram-positive and Gram-negative bacteria [14]. These peptides play a significant part in supporting bacteria within a bacterial cell community and typically demonstrate antimicrobial solid effects on other bacteria of similar or different genera [15]. 

## 2. Classification

Bacteriocins are antimicrobial peptides produced by nearly all prokaryotic lineages via ribosomal synthesis to eliminate competing organisms. Bacteriocins derived from Gram-positive bacteria are generally more prevalent than those from Gram-negative bacteria and *Archaea* [16]. Bacteriocins are classified based on the cell wall type of producing organisms (Gram-negative and Gram-positive). Several bacteriocins produced by the *Archaea* domain representatives have also been characterized, such as halocins and sulfolobaceae [17].

### 2.1. Bacterial AMPs Produced by Gram-Negative Bacteria

Bacteriocins display a wide range of size, structure, and mode of action variations. *E. coli* and other enterobacteria have been the primary sources of characterized bacteriocins in Gram-negative bacteria [15]. Gram-negative bacteria produce four main classes of bacteriocins: colicins, colicin-like, phage-tail-like bacteriocins, and microcins. 

#### 2.1.1. Colicins

Colicins are high-molecular-weight (30–80 kDa) bactericidal proteins sensitive to proteases and heat. They are synthesized by most *E. coli* strains carrying a single colicinogenic plasmid. These compounds are widely studied and serve as model systems for investigating bacteriocins’ structures, functions, and evolution. Colicin synthesis occurs under stress and is lethal for the producing cells due to co-expression with lysis protein [18].

#### 2.1.2. Phage Tail-like

Phage tail-like bacteriocins, or tailocins, are substantial protein structures ranging from 20–100 kDa and composed of 8 to 14 distinct polypeptide subunits. These structures exhibit similarities to the bacteriophage tail modules. Tailocins are present in bacterial genomes as a cluster of genes spanning over 40 kbp. This genetic locus encompasses genes responsible for encoding structural proteins, assembly enzymes, chaperones, regulatory genes, and lysis cassettes, all of which function to release bacteriocins into the surrounding environment [19]. Bacteriocins of this category are categorized into two groups—R and F. R-type tailocins are evolutionarily linked to the tails of phages in the *Myoviridae* family and form a lengthy tube enclosed by a shell, with a complex basal plate featuring receptor-binding proteins (RBPs) at one end. On the other hand, F-type bacteriocins, which belong to the tails of phages in the *Siphoviridae* family, lack a shell.

#### 2.1.3. Microcins

Microcins, which are the third type of bacteriocins produced by Gram-negative bacteria, are peptides that have a low molecular weight (<10 kDa) and are highly stable. These peptides play a role in competitive interactions among members of the *Enterobacteriaceae* family. They can resist proteases, extreme pH, and temperature variations. The genes responsible for encoding microcins are found in plasmid clusters and, less commonly, in genomic DNA [15]. These gene clusters consist of a variable number of genes but have a consistent organization, typically including open reading frames for microcin precursors, secretion proteins, immune factors, and, in some cases, post-translational modification enzymes. Microcins are categorized into two classes based on the presence, nature, and location of modifications, gene cluster organization, and the sequence of leader peptides [14].

**Figure 3:**
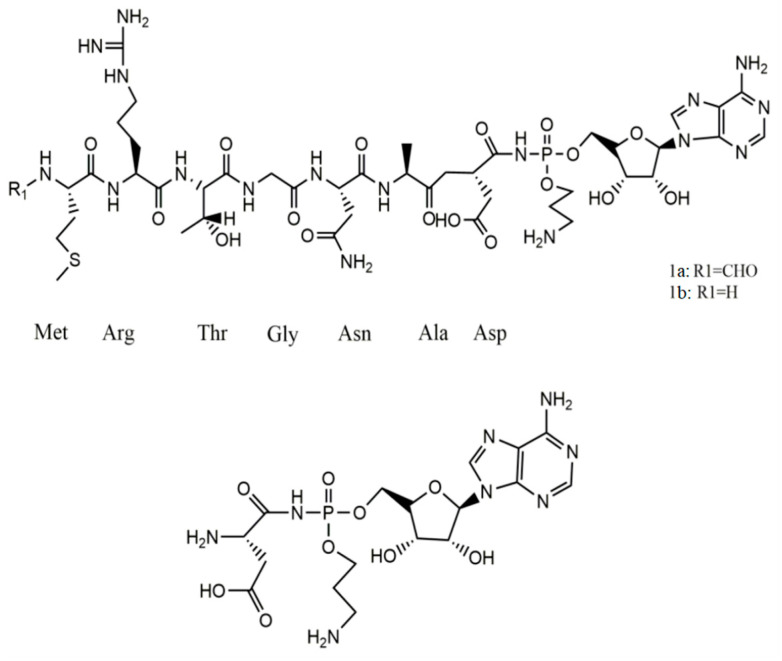
The structure of Microcin 7 (McC7). Intact McC7 (compound 1a), the deformylated variant (compound 1b), and processed McC7 (compound 2)) [22].

Peptides in class I have a molecular weight of less than 5 kDa. Examples of such peptides are microcins B17, C7-C51, and J25, which undergo complex post-translational modifications. Bacteriocins in this class can inhibit essential bacterial enzymes, including DNA gyrases I and II 68 S and RNA polymerases. Furthermore, they can impede the respiratory chain [20]. Class II comprises larger plasmid peptides (5–10 kDa) that do not undergo post-translational modifications. Examples include MccL, MccV, and Mcc24 and linear microcins encoded in the chromosome that carries a C-terminal siderophore, such as microcins E492, M, H47, I47, and G47. These peptides exert their toxic effects by creating pores and disrupting the cell membranes of the targeted pathogenic bacteria [21]. Figure 3 illustrates the structure of Microcin 7. Additionally, Table 1 provides details of significant research studies on antimicrobial peptides (AMPs) derived from bacteria.

The stability of AMPs in vivo is of great importance. It could be impacted by heat, pH, trypsin, pepsin, chymotrypsin, Proteinase K, and amylase. Darbandi et al. [23] depict detailed data about the stability of bacteriocins produced by the genera *Lactobacillus*, *Lactococcus*, *Enterococcus*, *Streptococcus*, *Pediococcus*, and *Carnobacterium*.

### 2.2. Bacterial AMPs Produced by Gram-Positive Bacteria

The majority of bacteriocins produced by Gram-positive bacteria fall into two primary categories. Lantibiotics and small post-translationally unmodified bacteriocins belong to Class I and II, respectively. Lantibiotics are peptides that undergo extensive post-translational modifications and contain lanthionine residues and methyllanthionine. In the last ten years, it has become evident that this group of ribosomally synthesized and post-translationally modified peptides (RiPPs) is more varied [17]. Historically, the third category of bacteriocins consisted of large (exceeding 10 kDa) antibacterial proteins and bacteriolysins, as well as tailocins of Gram-positive bacteria, which have been identified more recently.

#### 2.2.1. Lantibiotics

Lantibiotics are small peptides with a molecular weight of less than 5 kDa. They contribute to the structural firmness of bacteriocins and provide resistance against the actions of proteases. Lantipeptides are categorized into four classes based on their biosynthesis, two containing compounds with antibacterial properties. Three types of lantibiotics—AI, AII, and B—are identified by their structural characteristics, representing bacteriocins with linear, globular, and combined conformation, respectively [24]. Gram-positive bacteria produce two main types of bacteriocins: lantibiotics (class I) and non-lantibiotics (class II). Lantibiotics are short peptides consisting of 19–38 amino acids and undergo post-translational modifications, including thioether-based ring structures known as lanthionine or beta-methyllanthionine [25]. Some lantibiotics may also contain unique modified amino acids, such as D-alanine in lactocin *S. Penisin*, isolated from *Paenibacillus ehimensis*, shows activity against methicillin-resistant *Staphylococcus aureus* (MRSA). Unlike other lantibiotics, it effectively inhibits the growth of Gram-negative bacteria [26]. Due to their diverse structures, it is challenging to classify lantibiotics into subclasses. About 11 subclasses have been proposed for lantibiotics. Nisin, subtilin, lacticin 3147, and thuricin CD belong to class I, with nisin being the most extensively studied [27].

**Figure 4:**
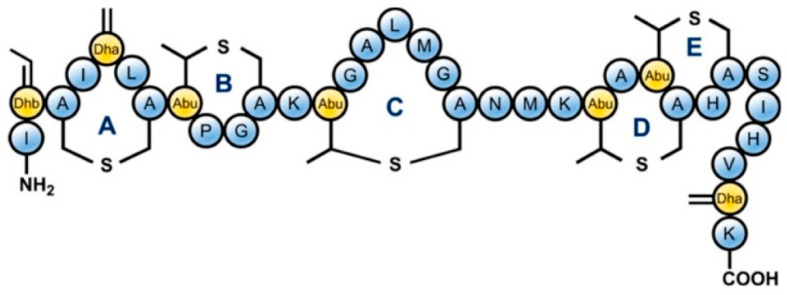
Structure of Nisin [29]. Dhb = dehydrobutyrine, Dha = dehydroalanine and Abu = aminobutyric acid.

Nisin, epilancin 15X, and microbisporicin from AI-type lantibiotics exert their antibacterial effects by inhibiting cell wall synthesis of the targeted cells through the binding of the bacteriocin N-terminal domain to lipid II, a precursor of peptidoglycan. The structure shown in Figure 4 depicts Nisin. Additionally, the C-terminal domain contributes to the formation of pores, disrupting the membrane potential. Mersacidin, actagardin, and cinnamicin belong to a type-B lantibiotic group characterized by a compact globular tertiary structure [28]. Bovicin HJ50 is assumed to bind to lipid II via its N-terminal fragment, with the C-terminal domain interacting with the membrane and the disulfide-containing region, forming a hairpin-like structure that disrupts the integrity of the phospholipid bilayer. A distinct group of lantibiotics, known as two-component lantibiotics, demonstrate synergistic antibacterial activity. Lacticin 3147, the profoundly studied two-component lantibiotic, is composed of type B α-peptide (LtnA1) and type A1 β-peptide (LtnA2). Its antibacterial effect also involves the formation of pores in the target cell membrane. Lantibiotics are of significant interest due to their structural diversity and potent activity against Gram-positive pathogens. Nisin, for instance, has been utilized as a natural food preservative for the past 50 years, while several new lantibiotics are undergoing clinical trials as antimicrobials.

Lantipeptides, such as lipolantins, are newly discovered and have antibacterial properties. These compounds are identified by the avionin residue and the N-terminal guanidino fatty acid [30]. Microvionin, a bacteriocin from *Microbacterium arborescens* 5913, is an essential member of this group. It is effective against MRSA and *Streptococcus pneumonia*, but the exact mechanism of action of lipolantins is not fully understood.

#### 2.2.2. Thiopeptides

Thiopeptides belong to class I and have diverse biological activities, including antibacterial, antiviral, and antiparasitic effects. Firmicutes and Actinomyces species are primary producers of thiopeptides. They disrupt protein synthesis by binding to the 50S ribosome subunit or elongation factors [31]. While thiopeptides are highly potent in the nanomolar range, their poor water solubility and low bioavailability limit their clinical use. GE2270A, a thiopeptide derivative, is currently under clinical trials for treating gastrointestinal infections caused by *Clostridium difficile*.

The Man-PTS, the main mannose permease in bacteria, serves as a receptor for class IIa bacteriocins (pediocin-like group) and class Iid lactococcin A (LcnA) and lactococcin B (LcnB). Class Iia bacteriocins are effective against *Clostridium difficile*, while LcnA-like bacteriocins only act against *Lactococcus lactis* strains. Garvicin Q (GarQ), a class Iid bacteriocin, shows little resemblance to LcnA-like bacteriocins and has a relatively broad antimicrobial spectrum, including activity against *Clostridium difficile* and *Lactococcus* spp., among others. 

#### 2.2.3. Modified Thiazole/Oxazole-Microcins-Boromycins

A cluster of bacteriocins with a similar structure to thiopeptides is altered thiazole/oxazole-microcins-boromycins. Their distinct characteristics encompass a macrocyclic amidine, decarboxylated C-terminal thiazole, and various uncommon β-methylated amino acid residues. The bottromycins, which have been discovered so far, are produced by bacteria of the genus *Streptomyces* and serve as potent agents against multidrug-resistant microorganisms such as MRSA and vancomycin-resistant enterococci (VRE) [32]. Additionally, bottromycins hinder protein synthesis by interacting with the bacterial 50S ribosomal subunit.

The altered thiazole/oxazole-microcins family also encompasses linear azole-containing peptides (LAPs). Currently, two compounds from this category—plantazolicin and goadsporin—have been structurally identified. Plantazolicin is a metabolic byproduct of *Bacillus amyloliquefaciens* FZB42, exhibiting selective antibacterial activity against closely related strains of the genus *Bacillus*. Goadsporin, derived from *Streptomyces* spp. TP-A0584, likewise demonstrates a limited spectrum of action, targeting only members of the genus. The mechanism of action of LAPs has not been investigated.

#### 2.2.4. Sactibiotics

Sactibiotics produced by the *Bacillus* genus comprise unique sulfur-containing peptides. These peptides possess antibacterial properties and can act as spermicides and hemolytics. The initial compound of this kind, subtilosin A, was discovered over 30 years ago. Sactibiotics are characterized by rigid hydrocarbon skeleton structures, with their amino acid residues positioned on the surface of spirals carrying a negative charge at a neutral pH, rendering them hydrophobic [33]. The absence of a common cationic charge suggests that sactibiotics are not drawn to the membrane but instead interact with specific receptors on or within the membrane due to their narrow spectrum of action. Research has observed that subtilosin A uses Trp34 as a membrane anchor, leaving anionic amino acid residues on the surface. Moreover, the peptide can partially integrate into the membrane, disrupting the membrane’s phosphate head groups and methylene groups of lipids. The extent of this disruption depends on the concentration of the bacteriocin, which results in the release of vesicular components. Sactipeptides, also known as sactibiotics, constitute a small subfamily of peptides.

**Figure 5:**
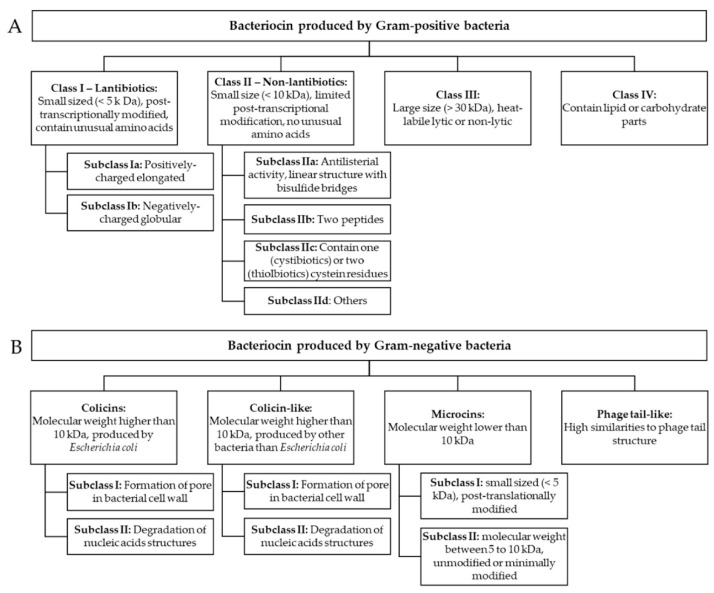
Classification of bacteriocins produced by Gram-positive (**A**) and Gram-negative (**B**) bacteria [12].

Sactipeptides, sulfur-to-alpha carbon thioether cross-linked peptides, are bacteriocins in the class of RiPPs. They exhibit diverse biological activities, including antibacterial and hemolytic properties [6]. Currently, six sactipeptides have been identified: subtilosin A, the sporulation killing factor SKF, thurincin H, thuricin CD (comprising the sactipeptides Trn-α and Trn-β), huazacin (also known as thuricin Z), and ruminococcin C. *Bacillus* strains are responsible for producing subtilosin A, thuricins, and SKF. Ruminococcin C is the only sactipeptide identified from a genus other than *Bacillus*; it was isolated from the human fecal strain *Ruminococcus gnavus* E1. While initially categorized as sactipeptides, thermocellin (CteA) and Tte1186a were recently reclassified as ranthipeptides, a type of ribosomally synthesized and post-translationally modified peptides distinguished by radical non-α-carbon thioether modifications [34]. Figure 5 presents the classification of bacteriocins produced by Gram-positive and Gram-negative bacteria.

### 2.3. Comparison of Differences between Bacteriocins Produced by Gram-Positive and Gram-Negative Bacteria

Bacteriocins produced by Gram-positive and Gram-negative bacteria differ significantly in structure, mode of action, target spectrum, and immunity mechanisms. Gram-positive bacteriocins are typically small peptides that are often cationic and amphipathic, which allows them to interact with bacterial membranes [35,36]. In contrast, Gram-negative bacteriocins, such as colicins and microcins, are larger proteins or small peptides [35,37]. The mode of action of Gram-positive bacteriocins generally involves membrane disruption, such as pore formation or targeting of lipid II, which leads to bacterial cell death [35]. On the other hand, Gram-negative bacteriocins typically enter the target cell via receptor-mediated mechanisms and either form ion channels or degrade DNA/RNA [38,39].

Regarding the target spectrum, Gram-positive bacteriocins tend to have broad activity against other Gram-positive bacteria. In contrast, Gram-negative bacteriocins often exhibit a narrow spectrum, targeting specific Gram-negative strains [35,36,37]. For immunity, Gram-positive bacteria produce immunity proteins that prevent self-targeting by neutralizing their own bacteriocins [35]. Similarly, Gram-negative bacteria produce immunity proteins that bind to the active domains of their bacteriocins, preventing self-killing [35,38,39,40]. These differences highlight the diversity of bacteriocin mechanisms across bacterial species, making them versatile tools for microbial control and therapeutic applications. In Table 2 are shown some differences between Gram-positive and Gram-negative bacteriocins.

## 3. Sources

Most bacteriocins currently used are obtained from the secondary metabolism of lactic acid bacteria (LAB). LAB is responsible for the lactic acid fermentation that turns lactose-rich milk into sour yogurt and represents valuable probiotics essential for human health due to the synthesis of many bioactive substances. LAB, which is a diverse group of Gram-positive asporogenic heterotrophic bacteria belonging to the phylum *Firmicutes*, has a long history of safe use and has a Generally Recognized as Safe (GRAS) and Qualified Presumption of Safety (QPS) status [41]. LAB produces substances that fit into all three categories of bacteriocins of Gram-positive bacteria. Additionally, there is a strong emphasis on studying microorganisms that make up the microbiota of humans and animals. The majority of bacteria present in the gut are capable of producing the necessary bacteriocins to maintain the stability of the microbial community. Typically, they harm pathogenic enterobacteria and actinomycetes [42].

Strains within the *Bacillus* genus generate numerous antimicrobial peptides with diverse chemical compositions. They can produce antimicrobial compounds such as peptides, lipopeptides, and bacteriocins. Similarly, *Bacillus* species are responsible for the production of primary antibiotics via ribosomal (bacteriocins) or non-ribosomal (polymyxins and iturins) pathways based on their mode of action [43]. *B. subtilis* produced the most antibiotics, followed by *B. brevis*, with a few produced by other *Bacillus* species [44]. Different strains of *B. subtilis* produce a variety of bacteriocins. For example, *B. subtilis*, *B. subtilis* A1/3, *B. subtilis* 168, *B. subtilis* strain HILY-85 produces subtilin, ericin S and ericin A, sublancin 168, mersacidin, respectively. Other *Bacillus* species, like *B. licheniformis*, *B. cereus*, *B. thuringiensis*, and *B. pseudomycoides*, etc. also produce bacteriocins like bacillocin 490, cerein 8A, thuricin 7, and pseudomycoicidin respectively. A new bacteriocin, amylocyclicin, was recently reported, produced by *B. amyloliquefaciens* FZB42 [45]. Sonorensin is a new peptide belonging to heterocycloanthracin, a subfamily of bacteriocin isolated from marine bacteria *B. sonorensis* MT 93 [46]. This peptide showed activity against broad-spectrum bacteria, including *B. subtilis*, *E. coli*, *Listeria monocytogenes*, *Pseudomonas aeruginosa*, *Staphylococcus aureus*, and *Vibrio vulnificus* [28].

The table below (Table 3) presents some of the bacteriocins mentioned in the review with their amino acid sequences.

**Figure 6:**
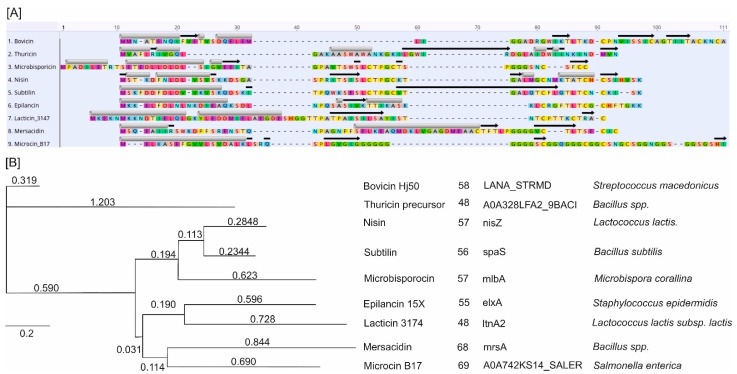
(**A**) Alignment of nine bacterial antimicrobial peptides (AMPs). The alignment was performed using Geneious Prime 2023.1(GraphPad Software LLC d.b.a. Geneious, Boston, MA, USA). Annotation revealed the distribution of alpha-helical domains, represented by grey cylinders above each peptide sequence, and beta-sheets depicted by black arrows. (**B**) Phylogenetic tree of the AMPs (Geneious Prime 2023.1). The tree includes the number of mutations per site, the length of the sequences, the corresponding genes, and their species of origin; the number of residue changes per site represents distance.

Figure 6A shows that whereas the amino acid sequences of the aligned peptides exhibit high variability, a general structural pattern is observed. This pattern is characterized by an enrichment of hydrophobic alpha helices at the N-terminus of the AMPs. This is associated with their ability to penetrate the microbial cell membrane and form pores. In contrast, the C-terminus is predominantly composed of beta-sheet structures, which can serve as structural elements and are also involved in membrane interactions, protein-protein interactions, and catalytic activity. More flexible loop regions were also presented and associated with the catalytic activity of the AMPs. Phylogenetically, Figure 6B illustrates the evolutionary relationships between the AMPs, with branch lengths indicating the number of mutations per site. The associated table provides additional information, including the number of amino acids, gene names, and species of origin for each AMP. The phylogenetic analysis reveals the genetic diversity and evolutionary distances among the AMPs, highlighting the mutations that have accumulated over time. Bovicin HJ50 and Thuricine precursor showed the highest divergence and formed two outgroups. Other AMPs showed relatively close relationships, forming three distinctive clusters: Nisin, Subtilin, Microbisporocin; Epilancin 15X and Lacticin; and Mersacidin and Microcin.

## 4. Mechanisms of Action

Ribosomally produced antimicrobial peptides are a varied collection of biologically active bacterial molecules that defend against other microorganisms [47]. Despite differences in their primary structure, their positively charged and amphiphilic properties allow them to attack target bacterial cells by disrupting the cell membrane [48]. 

AMPs primarily act by disrupting microbial cell membranes, leading to leakage of cellular contents and cell death through mechanisms like pore formation and membrane destabilization [49,50]. Some AMPs can also penetrate the cell and inhibit intracellular processes, such as DNA, RNA, or protein synthesis, amplifying their antimicrobial effects [51]. Additionally, AMPs possess immunomodulatory properties, enhancing the host immune response by recruiting immune cells and modulating inflammation [52]. They are also effective in disrupting biofilms, often resistant to conventional antibiotics, making them valuable in treating persistent infections [53,54].

**Figure 7:**
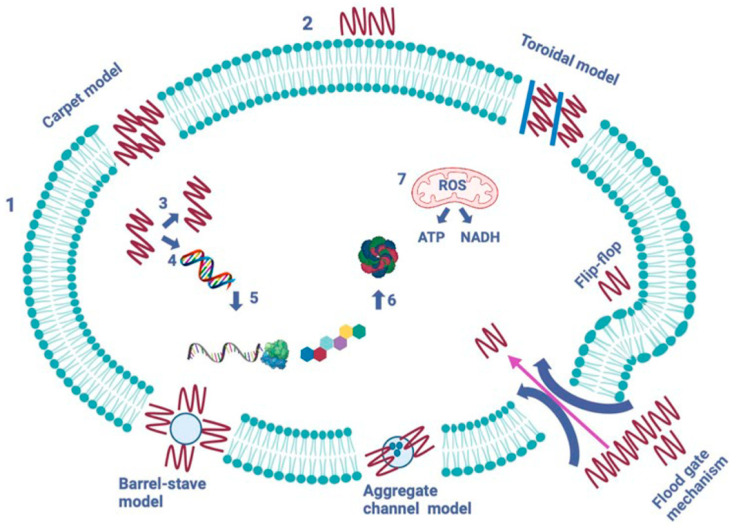
The mechanisms of action of AMPs on a pathogenic cell. The figure shows two significant types of peptides—membrane-bound and intracellular active peptides. The outer layer, “1”, is the cytoplasmic membrane of a pathogen cell, and the AMPs are marked as “2”. Membrane-bound peptides can act through five mechanisms: the barrel-stave, toroidal, carpet, floodgate, and aggregate channel models. The intracellular AMPs are represented by numbers “3” to “7” and show the inhibition of enzymes required for the binding of cell wall structural proteins, DNA and RNA synthesis, ribosomal functions and chaperone protein synthesis, and cellular respiration via ROS (Reactive Oxygen Species) formation and ATP (Adenosine Triphosphate) and NADH (Nicotinamide Adenine Dinucleotide) [6].

AMPs use five mechanisms to form pores and channels in the pathogen’s cytoplasmic membrane [55]. Peptide-induced pore generation aims to enable the leakage of cytoplasmic compartments through the lipid bilayer. In the barrel-stave model (proposed in 1977), peptides create trans-membrane pores by targeting the lipid core of the cytoplasmic membrane. The toroidal model (proposed in 2001) acts as a cascade aggregation of peptides that pierce the cell membrane multiple times. The aggregated peptides initiate other peptides to target and bind to the lipid monolayer, thus generating more membrane pores. Lacticin-Q, produced by lactococcal bacteria, uses the toroidal model. In the carpet model (proposed in 1992), AMPs act as a detergent, solubilizing and weakening the cytoplasmic membrane and giving it a carpet-like structure. According to the aggregate channel model (proposed in 2012), AMPs surround the membrane as spontaneously formed peptide aggregates, which create channels that allow cytoplasm containing all cell organelles to flow out. In the floodgate mechanism (proposed in 2011), α-helical AMPs, early in their attack, create temporary toroidal holes in the cell membrane. Next, these peptides attract neighboring unbound peptides, which are also involved in further cytoplasmic membrane hole formation [55]. Figure 7 summarizes the five known mechanisms of action of AMPs on pathogenic cells. 

Colicins are classified into three main groups based on their interaction mechanism with the target cell—pore-forming, nuclease, and peptidoglycan degrading. Receptors responsible for transporting nutrients such as vitamin B12 (cobalamin receptor BtuB), siderophore iron-binding FhuA, FepA, Cir, and Fiu, and nucleosides (Tsx receptor) facilitate the uptake of colicins by the target cell. Furthermore, certain colicins utilize porin proteins that regulate the passive diffusion of sugars, phosphates, and amino acids through the outer membrane (OM) [48]. Protein bacteriocins produced by other Gram-negative bacteria are deemed colicin-like due to their comparable structural and functional characteristics.

The precise method by which tailocins work is not entirely understood, but it likely involves compressing the shell and infiltrating the nucleus through the cell wall. This process creates a channel or pore that impacts the membrane potential of the target cell [19]. The most researched phage tail-like bacteriocins are R- and F-pyocins from *P. aeruginosa*. Tailocins generally have a limited bactericidal range, affecting specific subgroups of strains within the producing species.

Microcins possess potent antibacterial properties, which rely on intricate mechanisms to penetrate the outer and inner cell membranes as well as the cell walls of Gram-negative bacteria. To bypass the OM, siderophore microcins attach to receptors that are involved in iron transport. Cyclic microcin J25, distinguished by its N-terminal macrolactam ring, utilizes the hydroxamate receptor and the intracellular membrane protein SbmA. Meanwhile, Microcin C, produced as a heptapeptide adenylate, depends on external membrane porins and ABC membrane transporters. Once in the cytoplasm, it transforms into a non-hydrolyzable aspartyl-adenylate analog [56]. Despite employing different mechanisms to destroy target cells without any structural similarity, microcins adopt a common “Trojan horse” strategy, which could be harnessed in designing and creating new, effective antibiotics.

Nisin, a 34-amino acid lantibiotic, is produced by Gram-positive bacteria such as *Lactococcus*, *Staphylococcus*, and *Streptococcus* species. Various forms of nisin have been identified, including nisin A, nisin Z, nisin Q, nisin U, nisin F, nisin H, nisin O, nisin J, and nisin P. Nisin possesses antibacterial properties against a broad range of Gram-positive bacteria, including staphylococci, streptococci, enterococci, bacilli, and listeria. It binds to lipid II, an essential membrane component for peptidoglycan biosynthesis, leading to membrane permeabilization and inhibition of cell wall synthesis in targeted cells [57]. Moreover, in an MRSA model, nisin has been found to induce cell shrinkage and chromosomal DNA condensation, indicating interference with DNA replication or segregation in bacteria.

Epidermin, a 21-amino acid lantibiotic produced by *S. epidermidis*, demonstrates antimicrobial activity against staphylococci and streptococci. Its mechanism of action involves inhibiting cell wall synthesis through interaction with the cell wall precursor lipid II and sometimes by inducing pore formation [58]. In an in vitro catheter colonization model, epidermin significantly reduced *S. epidermidis* cells attached to silicone catheters. It also displayed antibacterial activity against over 85% of tested *S. aureus* (165 strains) responsible for bovine mastitis. In a separate study, epidermin exhibited antibacterial activity against 81.3% of tested *S. aureus* involved in human infections, including MRSA endemic clones in Brazil. Furthermore, epidermin demonstrated antibacterial effects against *S. hemolyticus*, *S. capitis*, *S. simulans*, *S. saprophyticus*, *S. hominis*, and *S. epidermidis*, although it showed no activity against certain tested *S. aureus*.

**Figure 8:**
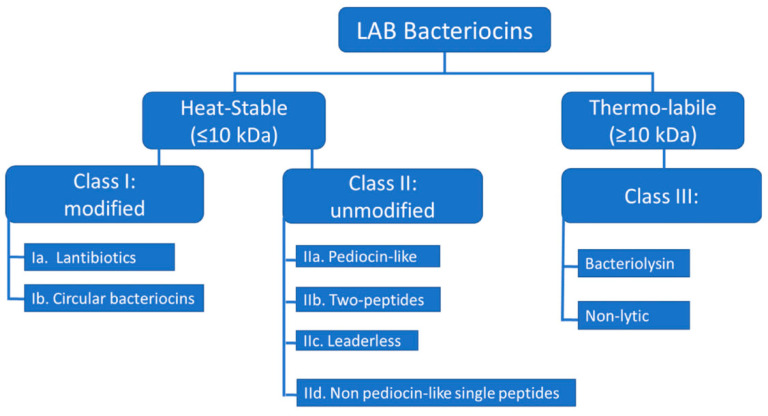
Suggested classification scheme for LAB-bacteriocins [60].

The most thoroughly researched LAB-bacteriocins can possess narrow spectra, acting solely on a limited range of target bacteria, typically within the same species, or have broad spectra targeting other species. There has been extensive research on the mode of action of LAB-bacteriocins against Gram-positive bacteria [59]. The number of LAB-bacteriocins with activity against Gram-negative bacteria is limited, unlike those with activity against Gram-positive bacteria. Although a small number of LAB-bacteriocins active against Gram-negative bacteria have been reported in the past decade, their mode of action is yet to be elucidated. LAB-bacteriocins’ effectiveness against Gram-negative target bacteria can be attributed to the structure of the cell envelope, which comprises three layers. The cytoplasmic membrane of Gram-negative bacteria is surrounded by an OM, consisting of a phospholipid bilayer and a network of lipids and polysaccharides known as lipopolysaccharides (LPSs) [60]. The OM phospholipids are linked to the inner leaflet of the membrane, and LPS is bound to the outer leaflet, known to cause endotoxic shock. It’s worth noting that LPS acts as a barrier to many antibiotics and hydrophobic compounds. However, LPS is the target of colistin, a polycationic antibiotic from the polymyxins group, which are cyclic non-ribosomal polypeptides (NRPs). Colistin is known to bind to LPS and phospholipids in the OM of Gram-negative bacteria, leading to leakage of intracellular contents and bacterial death. The rising number of Gram-negative pathogens resistant to fluoroquinolones, aminoglycosides, and β-lactams (carbapenems, monobactams, cephalosporins, and broad-spectrum penicillins) has led to the revival of colistin as a last resort therapeutic option for treating infections caused by Gram-negative bacteria that are resistant to the drugs mentioned above [61]. The classification of LAB-bacteriocins is exposed in Figure 8.

AMPs created by *Bacillus* spp. have become a hopeful substitute for antibiotics because of their wide-ranging ability to fight against resistant pathogens. Despite their potential, the limited production of AMPs under standard laboratory conditions remains a challenge for large-scale production [62].

The large-scale production of AMPs derived from *Bacillus* species faces several challenges. One key challenge is the low yield of AMPs, as they are often produced in limited quantities by native *Bacillus* strains, which hinders their economic feasibility for large-scale production [63]. To address this, genetic engineering techniques, such as overexpressing AMP-related genes and optimizing promoter strength, can enhance production [64]. Another challenge is the high cost of fermentation and purification, driven by complex growth media and stringent processing conditions [65]. This can be mitigated by using inexpensive substrates like agricultural by-products and optimizing fermentation parameters, as well as improving purification techniques like membrane filtration [65,66]. AMP stability is another concern, as AMPs can be unstable due to proteolytic degradation or unfavorable environmental conditions during production and storage [49]. Stability can be improved through structural modifications, such as amino acid substitutions or encapsulation techniques like microencapsulation [49]. AMPs are also sensitive to degradation by proteases produced either by the host organism or contaminating microbes, reducing their yield and activity [65]. This issue can be addressed by co-expressing protease inhibitors or using protease-deficient strains [65].

Regulatory and safety issues present another obstacle, as AMPs intended for food or medical use must undergo complex and costly approval processes due to concerns about toxicity, allergenicity, and resistance development [49]. Conducting comprehensive studies and engineering AMPs to reduce toxicity and improve specificity can help meet regulatory requirements [49]. Scaling up production from lab-scale to industrial scale poses challenges in maintaining consistent product quality and yield in large bioreactors [66,67]. Optimizing bioprocess parameters and using computational modeling can help ensure consistent production at an industrial scale [66,67]. Ensuring high purity and consistent quality of AMPs across production batches is difficult due to impurities and by-products from fermentation [63,64]. Robust quality control systems and advanced analytical techniques, such as mass spectrometry and HPLC, are crucial for ensuring product consistency. By addressing these challenges, *Bacillus*-derived AMPs have the potential for widespread use in medicine, agriculture, and food preservation [35,36,37].

Below is a combined table (Table 4) that includes all the challenges, potential solutions, and respective references for large-scale production of AMPs derived from *Bacillus* species.

Along with medium optimization and genetic manipulation, different molecular approaches have been examined to enhance the production of recombinant AMPs. These approaches involve selecting suitable expression systems, modifying expression promoters, and metabolic engineering. AMPs derived from *Bacillus* show significant promise as alternative antimicrobial agents [69].

## 5. Effect on Human Health

Bacteriocins’ primary uses in human health are essential, with one critical application being the utilization of bacteriocin-producing organisms as probiotics [70]. Probiotics refer to non-pathogenic and non-toxic strains that benefit humans and host animals. They can survive and maintain metabolic activity in the intestinal environment, remaining stable and viable for extended storage periods. Probiotics have shown potential in terms of antimicrobial production, competitive pathogen destruction, competition for nutrients, and modulation of the immune system. 

Probiotic strains play a crucial role in human health by producing bacteriocins that help maintain balance in the host microbiota and protect against pathogenic infections. Bacteriocins produced by probiotics, such as *Lactobacillus* and *Bifidobacterium* species, inhibit harmful bacteria in the gastrointestinal tract, promoting gut health and preventing infections [35,71]. These bacteriocins can also modulate the immune system, enhancing the body’s natural defense mechanisms. Probiotics and bacteriocins often work synergistically; while bacteriocins directly inhibit pathogens, probiotics compete for nutrients and adhesion sites, providing enhanced protection. However, potential concerns include antagonistic or toxic interactions, where certain bacteriocins might disrupt beneficial microbial populations or provoke adverse immune responses. Furthermore, some individuals are susceptible to allergic reactions, as certain bacteriocins may act as allergens, leading to immune hypersensitivity. Careful selection of strains, dose management, and monitoring for allergic reactions are critical for minimizing these risks [38,72].

Currently, numerous probiotics are utilized daily, including lactic acid bacteria, non-pathogenic strains of *E. coli*, *Bacilli*, and yeast [73]. Studies have indicated that purified bacteriocins or bacteriocin-producing probiotics can reduce pathogen numbers or alter intestinal microbiota composition in mice, chickens, and pigs. For example, a strain of *Lactococcus lactis* that produces nisin can promote the growth of *Bifidobacterium* and inhibit the growth of enterococci and streptococci in the intestines of rats across different regions such as the duodenum, ileum, caecum, and colon. Additionally, bacteriocins like Colicin Ib, E1, and microcin C7, which are derived from the *E. coli* H22 strain, demonstrate the ability to inhibit the growth of both pathogenic and non-pathogenic bacteria, including *Enterobacter*, *Escherichia*, *Klebsiella*, *Morganella*, *Salmonella*, *Shigella*, and *Yersinia*. Enterocin is a type of bacteriocin synthesized by the lactic acid bacteria *Enterococcus*. Enterocin has excellent antimicrobial effectiveness against foodborne pathogens, such as *L. monocytogenes* [74].

Furthermore, research has shown that probiotic strains of human origin, such as *Lactobacillus salivarius* UCC118, produce bacteriocin Abp118 that can eliminate *Listeria monocytogenes* cells. *Pediococcus* spp. Lactic acid bacteria are also well-documented as probiotics, and many *Pediococcus* strains produce pediocin, an effective agent for eradicating *Listeria* spp. [75]. Ongoing research aims to comprehensively study probiotics for potential use in the pharmaceutical and food industries. The MIC and inhibition diameter of some bacterial antimicrobial peptides are depicted in Table 5.

## 6. Limitations and Perspectives

Bacterial AMPs are vital in addressing the increasing antibiotic resistance of various pathogens, potentially serving as an alternative to antibiotics [76]. Bacteriocins have a narrow spectrum of action, making them highly specific antibacterial compounds that target particular bacterial pathogens [77]. With the wide range of natural bacteriocins available, it is relatively easy to identify effective drugs against specific human pathogens [56]. By developing and utilizing such narrow-spectrum antimicrobials, the number of available medications can be increased, and the lifespan of traditional antibiotics can also be prolonged [78]. 

Current research primarily focuses on the potential use of Gram-negative bacterial bacteriocins, specifically colicins and pyocins, as antibiotics. Although recent in vivo experiments have shown promising results, there are still unresolved questions about their suitability as therapeutics [5,79]. The limitations in using bacteriocins as therapeutic agents can be summarized as follows: (a) specificity of bacteriocins, as their narrow spectrum of activity can limit their effectiveness to only certain bacterial strains, necessitating accurate pathogen identification [35]. (b) Stability is another critical factor, as bacteriocins must resist protease degradation and remain active in various physiological conditions [80]. (c) Delivery mechanisms also play a crucial role, as ensuring that bacteriocins reach the infection site at effective concentrations can be challenging, especially in systemic infections [38]. (d) Additionally, the development of bacterial resistance to bacteriocins, though less common than with traditional antibiotics, can still occur and limit their long-term effectiveness [39]. (e) Finally, immune response modulation by bacteriocins can influence their therapeutic potential, as some bacteriocins may enhance the host’s immune defenses, further contributing to their effectiveness [35].

One key issue is the limited availability of data on the effects of bacteriocins on patients in terms of toxicity or immune response [81]. However, existing information does not indicate any harmful or toxic effects on the body, except for the mortality of chickens treated with pyocins. However, it is unclear whether the preparation was cleared of endotoxins. Furthermore, more research on dosage regimens and the timing of bacteriocin administration is necessary [82]. Addressing this issue requires more comprehensive pharmacokinetic studies [83]. Key factors influencing the effectiveness of bacteriocins in treating multidrug-resistant bacterial infections are the narrow spectrum of bacteriocins’ activity, their stability, and resistance to protease action, delivery mechanisms to the infection site, the development of bacterial resistance to bacteriocins, the immune host response modulated by bacteriocins, which can decrease their therapeutic potential, and finally crucial is the bacteriocins’ toxicity to human cells. 

**Figure 9:**
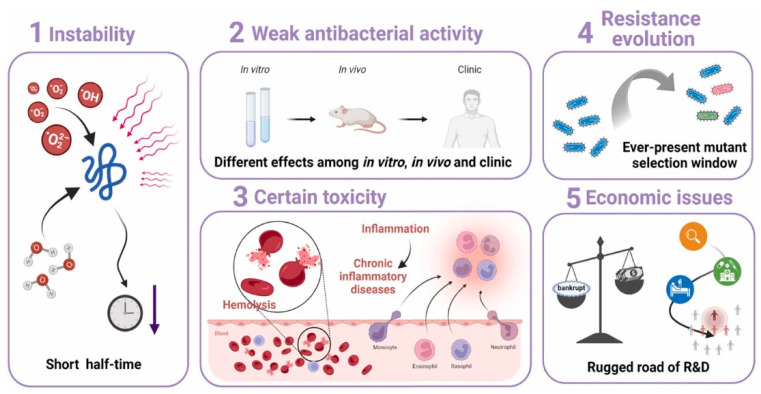
Challenges of drug-resistant AMPs. Reprinted with permission from [84]. 2024, Elsevier.

One disadvantage of bacteriocins is their toxicity to human cells. They interact with lipids in cell membranes disruptively, causing cell lysis. AMPs are hemolytic and unstable. Based on antibacterial mechanisms, AMPs with apparent toxic effects can be classified into (a) receptor-binding peptides and (b) membrane-active peptides. Instead of killing the target bacteria directly, receptor-binding peptides exert a bacteriostatic effect by modulating the host immune system. Sometimes, a high concentration of AMPs leads to inflammatory diseases such as dermatitis, rosacea, psoriasis, etc. [84]. Membrane-active peptides, such as pore-forming peptides, play hemolytic and cytotoxic properties. This goes via disassembling the membrane of eukaryotic cells, especially of erythrocytes, hence leading to hemolysis. Another side-effect of bacteriocins, for example, colicin, is damage to the kidney and the nervous system [85]. Figure 9 below depicts some challenges of drug-resistant AMPs.

On the other hand, the evolution of resistance against AMPs is lower than against conventional antibiotics. The dose-response curve of AMPs is steeper than that of conventional antibiotics due to the difference in pharmacodynamics (lower possibility of evolution of resistance) [86]. Polymyxin B and vancomycin are the AMPs currently approved for clinical application for drug-resistant bacteria.

The comparison of the efficacy of bacteriocins and conventional antibiotics has yielded promising results [87]. Piocin S5 is at least 100 times more effective than tobramycin in treating lung infections in mice, indicating its potential as a replacement for traditional antibiotics used against *P. aeruginosa*. However, most bacteriocins are known for their low stability and, therefore, need to be administered in higher doses with shorter intervals between doses [88]. This low stability, however, can also decrease the impact of the antibiotic on the body and the environment, ultimately minimizing the development of resistance [89]. Researchers have observed that environmental resistance to bacteriocins can arise by modifying cell surface receptors [90].

It is important to note that, despite the potential for resistance, many bacteria still respond to specific concentrations of bacteriocins. A new class of antimicrobial agents, termed ‘structurally nanoengineered antimicrobial peptide polymers’ (SNAPPs) exhibit activity against both Gram-positive and Gram-negative bacteria like *Enterococcus faecium*, *Staphylococcus aureus*, *Klebsiella pneumoniae*, *Acinetobacter baumannii*, *Pseudomonas aeruginosa*, and *Enterobacter* spp. (ESKAPE) and colistin-resistant and multidrug-resistant (CMDR) pathogens, while demonstrating low toxicity [91].

One disadvantage of using bacteriocins could be the innate resistance of the producing strains [92]. The presence of shared target cell destruction mechanisms and similar cytotoxic domains may lead to cross-immunity between bacterial strains. However, most bacterial strains either don’t produce immune proteins or only make a limited number [93]. As a result, bacteria can only develop resistance to a restricted range of bacteriocins. It is believed that using a combination of bacteriocins can overcome such resistance [94,95]. 

## Figures and Tables

**Table 1:** Table summarizing some of the bacterial AMPs with their source and activity.

Bacterial AMP	Source	Active against
Colicin	*E. coli*	*Enterobacter*, *Escherichia*, *Klebsiella*, *Morganella*, *Salmonella*, *Shigella*, and *Yersinia*
Gramicidin	*B. brevis*	Gram-positive; Gram-negative
Microvionin	*Microbacterium arborescens*	Methicillin-resistant *Staphylococcus aureus* (MRSA) and *Streptococcus pneumonia*
Plantazolicin	*Bacillus amyloliquefaciens*	Closely related strains of the genus *Bacillus*
Goadsporin	*Streptomyces* spp.	Closely related strains of the genus *Streptomyces*
Sonorensin	*B. sonorensis*	*B. subtilis*, *E. coli*, *Listeria monocytogenes*, *Pseudomonas aeruginosa*, *Staphylococcus aureus*, and *Vibrio vulnificus*
Nisin	*Lactococcus*, *Staphylococcus*, and *Streptococcus* spp.	Staphylococci, streptococci, enterococci, bacilli, and listeria
Epidermin	*S. epidermidis*	*S. hemolyticus*, *S. capitis*, *S. simulans*, *S. saprophyticus*, *S. hominis*, *S. epidermidis*, *S. aureus*
Microcin C7	*E. coli*	*Enterobacter*, *Escherichia*, *Klebsiella*, *Morganella*, *Salmonella*, *Shigella*, and *Yersinia*
Microcin L	*E. coli*	*Escherichia coli*, *Salmonella enterica*, *Shigella* spp., *Pseudomonas aeruginosa*
Abp118	*Lactobacillus salivarius*	*Listeria monocytogenes*
Pediocin	*Pediococcus* spp.	*Listeria* spp.
Bovicin HC5	*Streptococcus bovis*	*Listeria monocytogenes*
Bottromycin A2	*Streptomyces bottropensis*	Methicillin-resistant *Staphylococcus aureus* (MRSA), Vancomycin-resistant enterococci (VRE)
Enterocin A	*Enterococcus faecium*	*Listeria monocytogenes*

**Table 2:** Summary table of differences between Gram-positive and Gram-negative bacteriocins.

Aspect	Gram-Positive Bacteriocins	Gram-Negative Bacteriocins	References
Structure	Small peptides (often cationic, amphipathic)	Larger proteins (colicins) or small peptides (microcins)	[35,36,37]
Mode of Action	Membrane disruption (pore formation, lipid II targeting)	Receptor-mediated entry, followed by ion channel formation or enzymatic degradation (DNA/RNA)	[35,36,39,40]
Target Spectrum	Broad against Gram-positive bacteria	Narrow, usually targeting specific Gram-negative strains	[35,36,37]
Immunity	Immunity proteins prevent self-targeting	Immunity proteins bind to active domains, preventing self-killing	[35,36,37,39]

**Table 3:** Amino acid sequences of some bacteriocins and their respective UniProt identification numbers.

Bacteriocin	UniProt ID	Amino Acid Sequence
Nisin	P29559	MSTKDFNLDLVSVSKKDSGASPRITSISLCTPGCKTGALMGCNMKTATCHCSIHVSK
Colicin M	A0A761KWA3	METLTVHAPSPSTNLPSYGNGAFSLSAPHVPGAGPLLVQVVYSFFQSPNMCLQALTQLEDYIKKHGASNPLTLQIISTNIGYFCNAERNLVLHPGISVYDAYHFAKPAPSQYDYRSMNMKQMSGNVTTPIVALAHYLWGNGAERSVNIANIGLKISPMKINQIKDIIKSGVVGTFPVSTKFTHATGDYNVITGAYLGNITLKTEGTLTISANGSWTYNGVVRSYDDKYDFNASTHRGVIGESLTRLGAMFSGKEYQILLPGEIHIKESGKR
Microcin B17	A0A742KS14	MELKASEFGVVLSVDALKLSRQSPLGVGIGGGGGGGGGGSCGGQGGGCGGCSNGCSGGNGGSGGSGSHI
Subtilin	P10946	MSKFDDFDLDVVKVSKQDSKITPQWKSESLCTPGCVTGALQTCFLQTLTCNCKISK
Lacticin 3147	O87237	MKEKNMKKNDTIELQLGKYLEDDMIELAEGDESHGGTTPATPAISILSAYISTNTCPTTKCTRAC
Thuricin precursor	A0A328LFA2	MVAFLRIVGQLGAKAASWAWANKGKILGWIRDGLAIDWIINKINDMVN
Epilancin 15X	P86047	MKKELFDLNLNKDIEAQKSDLNPQSASIVKTTIKASKKLCRGFTLTCGCHFTGKK
Microbisporicin	W2EQT3	MPADILETRTSETEDLLDLDLSIGVEEITAGPAVTSWSLCTPGCTSPGGGSNCSFCC
Mersacidin	A0A2H4RAU1	MSQEAIIRSWKDPFSRENSTQNPAGNPFSELKEAQMDKLVGAGDMEAACTFTLPGGGGVCTLTSECIC
Bovicin HJ50	H2A7G5	MMNATENQIFVETVSDQELEMLIGGADRGWIKTLTKDCPNVISSICAGTIITACKNCA

**Table 4:** Challenges and potential solutions for large-scale production of AMPs derived from *Bacillus* species.

Challenge	Potential Solution	Reference
Low yield of AMPs in native *Bacillus* strains	Genetic engineering to enhance AMP production (e.g., overexpression, synthetic biology)	[63,64]
High cost of fermentation and purification	Develop cost-effective fermentation strategies (e.g., using inexpensive substrates, optimizing conditions) Improve purification techniques (e.g., membrane filtration, affinity chromatography)	[64]
AMPs can be unstable (e.g., proteolytic degradation)	Modify AMP structure (e.g., cyclization, amino acid substitution) to improve stability Use encapsulation techniques (e.g., microencapsulation, nanocarriers) to protect AMPs	[49,66,67]
Protease degradation of AMPs during production	Co-express protease inhibitors or use protease-deficient strains Produce protease-insensitive forms of AMPs	[49,68]
Regulatory approval challenges (e.g., toxicity, allergenicity)	Conduct comprehensive safety studies and improve AMP specificity Rational design of AMPs to reduce toxicity and improve specificity	[49,63,64]
Scaling up from lab-scale to industrial-scale	Optimize bioprocess parameters (e.g., nutrient feed rates, aeration, agitation) Use computational modeling and process control systems	[66,67]
Achieving high purity and consistent quality	Implement robust quality control systems (e.g., SOPs, batch consistency) Use advanced analytical techniques (e.g., mass spectrometry, HPLC, NMR)	[63,64,66]

**Table 5:** Antibacterial activities of various bacteriocins produced by Gram-positive and Gram-negative bacteria [12].

Bacteriocin	Producer Strain	Active against	MIC (mg/L)	Inhibition Diameter (mm)
Nisin A	*Lactococcus lactis*	MRSA	0.5–4.1	
		Vancomycin-intermediate *S. aureus* (VISA)	2 ≥ 8.3	
		VRE	2 ≥ 8.3	
Epidermin	*Staphylococcus epidermidis*	*Staphylococcus aureus*		>14
		*Streptococcus agalactiae*		>14
Bovicin HC5	*Streptococcus bovis*	*Listeria monocytogenes*		>16
Bottromycin A2	*Streptomyces bottropensis*	MRSA	1	
		VRE	0.5	
Pediocin PA-1	*Pediococcus acidilactici*	*Listeria monocytogenes*	0.0013–0.0062	
Enterocin A	*Enterococcus faecium*	*Listeria monocytogenes*	0.0002–0.0011	
Microcin L	*Escherichia coli*	*Escherichia coli*	12–18	
		*Salmonella enterica*	12–18	
		*Shigella* spp.	12–18	
		*Pseudomonas aeruginosa*	8–12	

## Data Availability

The data used during the current study are available from the corresponding author upon request.
